# A Journey of Cytolethal Distending Toxins through Cell Membranes

**DOI:** 10.3389/fcimb.2016.00081

**Published:** 2016-08-10

**Authors:** Kathleen Boesze-Battaglia, Desiree Alexander, Mensur Dlakić, Bruce J. Shenker

**Affiliations:** ^1^Department of Biochemistry, SDM, University of PennsylvaniaPhiladelphia, PA, USA; ^2^Department of Microbiology and Immunology, Montana State UniversityBozeman, MT, USA; ^3^Department of Pathology, SDM, University of PennsylvaniaPhiladelphia, PA, USA

**Keywords:** cytolethal distending toxin (CDT), cholesterol, CRAC site, phosphatases, PI3K pathway inhibitors

## Abstract

The multifunctional role of lipids as structural components of membranes, signaling molecules, and metabolic substrates makes them an ideal partner for pathogens to hijack host cell processes for their own survival. The properties and composition of unique membrane micro-domains such as membrane rafts make these regions a natural target for pathogens as it affords them an opportunity to hijack cell signaling and intracellular trafficking pathways. Cytolethal distending toxins (Cdts), members of the AB2 family of toxins are comprised of three subunits, the active, CdtB unit, and the binding, CdtA-CdtC unit. Cdts are cyclomodulins leading to cell cycle arrest and apoptosis in a wide variety of cell types. Cdts from several species share a requirement for membrane rafts, and often cholesterol specifically for cell binding and CdtB mediated cytotoxicity. In this review we focus on how host–cell membrane bilayer organization contributes to the cell surface association, internalization, and action of bacteria derived cytolethal distending toxins (Cdts), with an emphasis on *Aggregatibacter actinomycetemcomitans* Cdt.

## Introduction

Cell membranes play a critical role in the events involved in the interaction between host cells and microbial pathogens and/or their products. The host cell plasma membrane is a central element in pathogen-host associations with interaction(s) at the cell surface leading to internalization, while intracellular membranes play a critical role in subsequent pathogen trafficking. Ultimately, such pathogen-lipid interactions work coordinately to mediate cytotoxicity. Biological membranes primarily consist of (glycero)-phospholipids, sphingolipids, and cholesterol, with ~50% of the membrane volume comprised of transmembrane, peripheral, and lipid-linked proteins, all arranged in a bilayer configuration. Lipids not only provide the structural backbone of biomembranes, but also play a critical role in cellular signaling, membrane microdomain organization and dynamics, membrane trafficking as well as serving as energy storage molecules (for review see van der Meer-Janssen et al., 2010). Pathogens often take full advantage of the multi-functional role of lipids to modulate host cell processes in order to facilitate their own survival and replication. In this review, we summarize how membrane bilayer organization contributes to the cell surface association, internalization, and action of bacteria-derived cytolethal distending toxins (Cdts).

## Cytolethal distending toxins

Cytolethal distending toxins are members of a group of bacterial toxins termed “cyclomodulins” that interfere with the eukaryotic cell cycle, thereby inhibiting or at least interfering with the normal function of dividing cell populations. These toxins represent a family of potential virulence factors encoded by over 30 pathogenic γ and ε-proteobacteria (Gargi et al., 2012). The *cdt* genes are found in a diverse group of gram-negative bacteria that colonize different niches within the host. These include; *Aggregatibacter actinomycetemcomitans*, an oral pathogen; *Haemophilus ducreyi*, a genital pathogen; as well as diarrheal disease-causing gastric pathogens such as *Campylobacter jejuni*, and some *Escherichia coli* isolates (Pickett et al., 1994, 1996; Okuda et al., 1995, 1997; Comayras et al., 1997; Pickett and Whitehouse, 1999; Klionsky et al., 2016). For clarity we will utilize the nomenclature proposed by Thelestam (Thelestam and Frisan, 2004) where each CDT is specified by adding the initials of the bacterial source before Cdt, for example, *Aa*Cdt refers to *A. actinomycetemcomitans* Cdt.

Cdts are encoded by three genes, designated *cdtA, cdtB*, and *cdtC*, which are arranged as an apparent operon (Shenker and Gray, 1976; Shenker et al., 1999, 2000, 2001; De Rycke and Oswald, 2001; Nesic et al., 2004; Thelestam and Frisan, 2004). These three genes specify three polypeptides designated CdtA, CdtB, and CdtC with apparent molecular masses of 28, 32, and 20 kDa, respectively. Together the subunits form a functional heterotrimeric holotoxin of CdtA, CdtC, and CdtB in a 1:1:1 ratio. Cdt, a heterotrimeric holotoxin typically functions as an AB2 toxin where CdtB is the active (A) unit and the complex of CdtA and CdtC comprise the binding (B) unit (Elwell et al., 2001; Lara-Tejero and Galan, 2001; Nesic et al., 2004). It is universally accepted that Cdts are bacterial exotoxins which must gain access to the host cell's intracellular milieu to exert their cytotoxic effects. Thus it was anticipated that mode of entry is likely cell type specific given that the Cdt binding subunit (CdtA-CdtC) must interact with a cell surface receptor. Recent evidence suggests that this assertion is limiting because several Cdt binding subunits interact directly with cholesterol in the context of lipid rafts. The ubiquitous nature of membrane rafts and plasma membrane cholesterol suggests that cytotoxic specificity is likely not mediated by association of CdtA and CdtC with cell surface specific receptors but rather by downstream signaling and/or trafficking pathways which may be unique to individual cell types.

## Cdt association with the cell surface

Cdts are intracellular-acting toxins and they must first associate with the cell surface to exert their toxic effects; this critical interaction is followed by internalization and trafficking to their site(s) of action. Cell surface recognition involves the identification and association of toxin subunits with specific cell membrane moieties such as membrane protein, lipid and carbohydrate components. In the case of Cdt, surface association is dependent on the binding unit which is composed of the subunits CdtA and CdtC (Nesic et al., 2004; Thelestam and Frisan, 2004; McSweeney and Dreyfus, 2005; Yamada et al., 2006). Indeed, both *Aa*CdtA and *Aa*CdtC are required for maximal *Aa*CdtB internalization (Boesze-Battaglia, 2006; Boesze-Battaglia et al., 2006; Damek-Poprawa et al., 2012). However, it should be noted that recent studies suggest that *Hd*CdtA and *Hd*CdtC independently support *Hd*CdtB induced toxicity of HeLa and CHO-K1 cells; similar results were obtained with *Ec*Cdt (strain K12) (Dixon et al., 2015). Furthermore, *Aa*CdtAB was shown to intoxicate KB oral epidermal cells while *Aa*CdtBC did not (Saiki et al., 2001). In those studies, cell death was analyzed 72 h after high dose toxin treatment, at which point mechanistic differences between the different CdtAB and CdtBC and holotoxin would be masked. *Haemophilus parasuis* (*Hp*) encodes for two copies of cytolethal distending toxin. In contrast to the other bacterium, *Hp*CdtAB exhibited higher toxin activity than *Hp*CdtCB (Zhou et al., 2012).

Gram negative bacteria shed outer membrane vesicles (OMV) or bleb-like structures during normal growth. The release of OMVs allows for the delivery of virulence factors such as toxins in a compact package to the host cell without close contact with the bacteria. The mode of action of OMVs in bacterial virulence is not well-understood, however these structures may hijack normal communication pathways resulting in cell death. Proteomic profiling of OMVs isolated from *E. coli* identified periplasmic and outer membrane proteins, as well as the cytolethal distending toxin as vesicular components (Berlanda Scorza et al., 2008). *EcII*Cdt, *Cj*Cdt, and *Aa*Cdt are secreted encapsulated within outer membrane vesicles, with all three subunit comprising the holotoxin detected in the OMVs (Berlanda Scorza et al., 2008; Lindmark et al., 2009; Elmi et al., 2012; Murase et al., 2016). In the case of *A. actinomycetemcomitans*, OMVs may be used as a vehicle for delivery of *Aa*Cdt to human gingival fibroblasts and HeLa cells (Thay et al., 2014). Given that *A. actinomycetemcomitans* has been implicated in aggressive forms of periodontitis, the observation that OMVs contain biologically active CdtB suggests that this mode of delivery may promote damage in the sulcular/junctional epithelium (Thay et al., 2014). This proposed mode of action remains to be experimentally verified. An intriguing aspect of *Cj*OMV and *Aa*OMV uptake is the requirement for cholesterol rich membrane rafts in the fusion of OMVs with the host cell surface (Elmi et al., 2012; Rompikuntal et al., 2012). Once internalized, *A. actinomycetemcomitans, E. coli*, and *P. aeruginosa* OMV-specific components co-distribute with the endoplasmic reticulum. In the case of *Aa*OMV and *Pa*OMV, the delivery of the intra-vesicular cargo was not dependent on retrograde transport as inhibition of the transport pathway had no effect on OMV uptake (Rompikuntal et al., 2012). Although the role of OMVs in Cdt uptake is documented, the vast majority of binding and toxicity studies utilize individually synthesized subunits that are recombined to generate the holotoxin.

The requirement for the interaction of CdtA and/or CdtC with cell membrane components is consistent with the crystal structure of both *Aa*Cdt and *Hd*Cdt which show direct contact between CdtA-CdtB, CdtA-CdtC, and CdtB-CdtC. Based on the crystal structure of *Hd*Cdt the site of Cdt-cell surface interaction is predicted to involve two binding elements; an aromatic patch in CdtA and a deep grove at the interface of CdtA and CdtC (Nesic et al., 2004). In addition, CdtA and CdtC adopt a lectin-like structure, with structural homology to ricin, a plant toxin known to bind N-acetylgalactosamine or beta-1,4-linked galactose. The association of Cdts with a specific cell surface protein is based on indirect evidence: a haploid-genetic screen identified a putative G-protein coupled receptor, TMEM181, as a likely binding partner of Cdt. TMEM 181 mutants expressed in a myeloid leukemia cell line, KBM7, were resistant to the toxin, although no cell surface association or direct binding studies were undertaken (Carette et al., 2009).

Currently, there is no ubiquitous receptor for the species-specific Cdts. *Cj*Cdt, *EcII*Cdt, and *Aa*Cdt were all found to bind to HeLa cells (Lee et al., 2003; McSweeney and Dreyfus, 2005). *EcII*Cdt binding was dependent on the glycosyl group, fucose, with fucose specific binding proteins shown to block *EcII*Cdt mediated cell cycle arrest. Moreover, the *EcII*CdtA and *EcII*CdtC subunits bind to N-linked fucosyl glycoproteins such as thyroglobulin. This subunit-glycoprotein association prevented the *EcII*Cdt holotoxin from binding to HeLa cells. *Aa*CdtA also bound N-linked fucosylated glycoproteins *in vitro* (Cao et al., 2008). Binding to glycosylated residues was not limited to those associated with proteins: *Aa*CdtA and *Aa*CdtC both associated with the gangliosides (glycosyl-ceramides), GM1 and GM2, with *Aa*CdtA also exhibiting specificity for GM3 in a human monocytic cell line. Pre-incubation of U937 cells with a glycosyl-ceramide inhibitor rendered the cells resistant to *Aa*Cdt toxicity. Furthermore, pre-incubation of *Aa*Cdt with GM3 enriched liposomes resulted in decreased Cdt toxicity as measured by DNA damage (Mise et al., 2005).

In a comprehensive study, Eshraghi et al. (2010), compared the binding properties and intoxication profiles of *Cj*Cdt, *Hd*Cdt, *Aa*Cdt, and *Ec*Cdt in a series of diverse cell lines (Eshraghi et al., 2010). 3T3 fibroblasts as well as Y-1 adrenal cells were resistant to *Hd*Cdt, *Aa*Cdt, and *Ec*Cdt as measured by phospho-H2AX immunostaining, which localizes to double stranded DNA breaks; however, they were susceptible to *Cj*Cdt intoxication. *Hd*Cdt and *Aa*Cdts were more active on HeLa and CHO-K1 cells than *Ec*Cdt and *Cj*Cdt. Using a panel of well-characterized glycan deficient mutant CHO-K1 cells, the specificity for Cdt association with a N-, O-, or lipid-linked glycans was tested. Although no cell line displayed a resistance to any of the Cdts tested, deficiency in the N-linked glycans resulted in increased sensitivity to all four Cdts. Of note, CHO-K1 cells with deficiencies in sialic acid were found to be hypersensitive to both *Hd*Cdt and *Aa*Cdt. Whether these increases are due to a cell surface recognition event or to an intracellular trafficking defect remains to be determined. These studies provide no clear pattern for Cdt cell surface associations but rather highlight the heterogeneous nature of Cdt- host cell associations, with Cdts exploiting different cell surface molecules to gain entry.

## Cdt association with membrane domains

Commonality between the Cdts emerges when one expands the notion of binding partners to include cell membrane domains, specifically lipid micro-domains or membrane rafts. Membrane bilayer organization is determined by interactions between membrane proteins and lipids. For example, sphingolipids and cholesterol preferentially interact with each other resulting in a spontaneous separation of these lipids from other phospholipids in the cell membrane. Such a lateral phase separation is proposed to result in the formation of distinct domains that are less than 50 nm in diameter, although they can coalesce to form larger signaling platforms. These microdomains are mainly composed of (glyco-) sphingolipids and cholesterol as well as a specific subset of transmembrane proteins, or proteins peripherally associated with the bilayer through acyl or GPI-anchors. The selective exclusion or inclusion of transmembrane receptors as well as intracellular proteins within these domains is necessary for mediating numerous cellular processes including endocytosis, signaling, protein sorting, and intracellular membrane trafficking. The mechanisms that mediate the preferential partitioning of proteins into rafts or their complete exclusion from rafts are largely unknown. Biological functions as well as controversies surrounding the role of lipid rafts *in vivo* is discussed in several reviews and is beyond the scope of this review (Munro, 2003; Simons and Vaz, 2004; Shaw, 2006; Jacobson et al., 2007; Varshney et al., 2016).

The properties and composition of membrane rafts make these regions a natural target for pathogens as it affords them an opportunity to hijack and influence a number of cellular functions. For instance, the abundance of signaling molecules in these domains allows pathogens a mode of communication with the host cell, while distinct domains serve as endocytic platforms for internalization and ultimately trafficking to subcellular compartments. A critical biophysical property of these regions is their intrinsic ability to transiently oligomerize. Host-pathogen interactions can disrupt, stabilize or otherwise alter these platforms, resulting in alterations in host signaling, promotion of pathogen uptake, targeting of pathogens to intracellular pathogen vacuoles instead of host degradative pathways, and often contribute to pathogen egress (van der Meer-Janssen et al., 2010). Using a combination of these and other mechanisms, more than 100 pathogens have been suggested to interact with lipid rafts and hereby facilitate their pathogenicity for review see (Riethmuller et al., 2006).

Several components of lipid rafts as well as biophysical properties associated with these structures have been linked to Cdt binding and toxicity as shown in Table 1. The focus of this particular discussion will be centered on sphingomyelin, cholesterol, and glycolipids as Cdt “receptors.” *Cj*Cdt, *Hd*Cdt, *Aa*Cdt, and *Ec*Cdt all appear to require N-linked glycans for toxicity; such complexes are abundant in membrane rafts. Two common components of membrane rafts, sphingomyelin and cholesterol, are also intimately associated with Cdt binding and toxicity. The haploid genetic screen that identified TMEM181 also identified sphingomyelin synthase 1 (SGMS1) as required for *Ec*Cdt toxicity as measured by cell cycle arrest (Carette et al., 2009). A follow-up study using the KBM7 gene inactivation screening method expanded the requirement for sphingomyelin synthase to include *Aa*Cdt and *Hd*CDt as well as *Cj*Cdt (Carette et al., 2011). It is known that depletion of SGMS1 activity alters raft associated signaling complexes (Miyaji et al., 2005). In LY-B cell, a cell line deficient in sphingolipid biosynthesis, *Aa*Cdt had no cytotoxic effect (Mise et al., 2005). Further evidence in support of Cdt-raft association is the co-distribution of the Cdt holotoxin with GM1 and/or caveolin enriched membrane regions. Such co-localization was first described for *Aa*Cdt; confocal microscopy documented the association of all three Cdt subunits with GM1 enriched membrane rafts (Boesze-Battaglia, 2006). Subsequently, *Cj*Cdt was shown to co-distribute with caveolin-rich membrane rafts in CHO-K1 cells (Lin et al., 2011) with *Cj*CdtB binding dependent on the association of *Cj*CdtA and *Cj*CdtC with membrane rafts.

**Table 1:** **Summary showing the effects of membrane components on CDT toxicity in CHO-K1 cells**.

**Bacterial Species^†^**	**Impact on CDT Toxicity**				
	**N-linked glycan deficiency**	**Fucosylated N-linked glycan deficiency**	**Cholesterol enrichment**	**Cholesterol depletion**	**Sialic acid deficiency**
*Aa*	+	NC	+	ND	+
*Hd*	+	NC	+	ND	+
*Ec*	+	NC	+	−^*^	+
*Cj*	+	NC	NC	−	NC

*Intoxication was determined using propridium iodide staining to track G2/M cell cycle arrest. Conclusions taken from Eshraghi et al. (2010), except where indicated*.

* *+, increased toxicity; –, reduced toxicity; NC, no change; ND, not determined in CHO cells*.

† *Abbreviations for bacterial species (with specific strain) are as follows: Aa, Aggregatibacter actinomycetemcomitans (Y4); Hd, Haemophilus ducreyi (35000HP); Ec, Escherichia coli (S5); Cj, Campylobacter jejuni (81–176)*.

Numerous *in vitro* studies suggest that the structure of lipid rafts is stabilized in ice cold non-ionic detergents. Solubilization of cell membrane preparations with Triton X-1oo as well as several other detergents results in the isolation of detergent resistant membranes (DRMs) enriched in raft–associated components. DRMs have been isolated from *Aa*Cdt and *Cj*Cdt treated Jurkat and CHO-KI cells respectively (Boesze-Battaglia et al., 2006; Lin et al., 2011). In both cases, all three Cdt subunits were DRM associated; in the absence of CdtA and CdtC, CdtB did not associate with rafts. In the case of *Aa*Cdt treatment, two separate DRMs were reported which differed not only in the relative levels of *Aa*CdtB but also the levels of phosphoinositide lipids (Boesze-Battaglia et al., 2006), a relationship that will be discussed below.

Cholesterol, a major constituent of membrane rafts, is composed of a highly hydrophobic sterol ring system and a small 3-hydroxyl moiety. In contrast to sphingolipids, cholesterol is much smaller and does not contain a long acyl tail. Thus at 37°C, sphingolipids and cholesterol can segregate into specific microdomains in which the sphingolipid headgroups occupy a large volume with cholesterol acting as a spacer, filling voids between sphingolipids. To date, the majority of studies implicating Cdt binding and activity as requiring membrane rafts has focused on depletion or enrichment of cholesterol in cell membranes. Such studies have focused on the use of methyl-B-cyclodextrin (MβCD) to remove cholesterol from the plasma membrane prior to toxin treatment. Cyclodextrins are cyclic oligomers of glucose that have the capacity to sequester lipophiles in their hydrophobic core (Pitha et al., 1988). The water-soluble MβCD is known to form soluble inclusion complexes with cholesterol, thereby enhancing its solubility in aqueous solution (Pitha et al., 1988; Irie et al., 1992a,b). Initial studies utilizing MβCD-mediated cholesterol depletion showed loss of *Hd*Cdt intoxication measured as G2 arrest by flow cytometry in HeLa cells (Guerra et al., 2005). Furthermore, *HdCd*t surface binding decreased upon cholesterol depletion in these studies. Cholesterol depletion of lymphoid cells resulted in loss of *AaCdt* induced cell cycle arrest, an effect that was reversed upon cholesterol repletion of the plasma membrane (Boesze-Battaglia et al., 2006). MβCD-mediated loss of cholesterol in CHO-K1 cells did not alter *Aa*CdtA cell surface association (Damek-Poprawa et al., 2012). *HdCdt, AaCdt*, and *EcCdt* mediated cell cycle arrest was enhanced when CHO-K1 cells were cholesterol loaded (Eshraghi et al., 2010). In contrast, treatment of cholesterol-loaded cells (CHO-K1) with *CjCdt* did not enhance cell cycle arrest (Eshraghi et al., 2010). *Hp*Cdt dependence on cholesterol was cell type specific with loss of cholesterol in Hep-2/Vero cells resulting in increased G2 population, while in mouse epithelial cells (PAM cells) cholesterol depletion resulted in a dose dependent decrease in the G2 population (Zhou et al., 2012). It is important to note that the action of MβCD is not limited to cholesterol depletion, it also inhibits calcium mobilization and alters cell polarization (Pizzo and Viola, 2003). Thus to control for these effects cells should also be treated with cholesterol saturated MβCD. While this treatment does not result in cholesterol depletion, it does induce the other actions associated with MβCD. Such control studies were only performed for the *Aa*Cdt and *Cj*Cdt (Boesze-Battaglia et al., 2009; Ahmado et al., 2011).

The hydrophobic nature of the deep groove in the crystal structure of Cdt as well as the requirement for cholesterol to induce cell cycle arrest suggest that binding subunits, CdtA and/or CdtC, contain a cholesterol recognition motif (Epand, 2006). Several proteins bind cholesterol; these include the benzodiazepine receptor, the human immunodeficiency virus transmembrane protein gp41, and caveolin (Li and Papadopoulos, 1998; Vincent et al., 2002; Epand et al., 2005; Jamin et al., 2005). Each of these cholesterol-binding proteins contain the cholesterol recognition amino acid consensus sequence (CRAC), (L/V) X1–5YX1–5(R/K), where X1–5 represents one to five residues of any amino acid. Critical to cholesterol binding is the tyrosine residue of the CRAC motif: Jamin et al. mutated this residue within the benzodiazepine receptor, which resulted in a loss of cholesterol binding (Jamin et al., 2005).

Motif analysis of the “binding” subunits, CdtA and CdtC, identified a CRAC site within the CdtC subunit, 68-LIDYKGK-74. Structural analysis of CdtC in the context of the holotoxin indicates that this site is at the surface of the molecule and accessible to bind the membrane. The *Aa*CdtC subunit was shown to bind preferentially to cholesterol containing LUVs (Boesze-Battaglia et al., 2009). Mutation of the tyrosine residue within the CdtC CRAC motif also resulted in significant reduction in the ability of the holotoxin to interact with LUVs. Moreover, the mutant toxin exhibited reduced binding to Jurkat cells, mast cells, macrophages as well as HeLa cells along with a reduced intracellular transfer of CdtB, and a concomitant reduction in toxicity (Boesze-Battaglia et al., 2015). We propose that binding of cholesterol by the CRAC region contained in the CdtC subunit results in the association of the Cdt holotoxin with membrane lipid rafts. These studies suggest that in a manner analogous to other CRAC site containing proteins which sequester cholesterol, the CdtC subunit may not only target the holotoxin to a raft domain but also stabilize the association of the toxin with this domain. This stable association has important consequences for Cdt induced toxicity; it generates toxin rich regions that remain active until cholesterol is removed or toxin is internalized.

It is likely that CdtC lipid raft association is critical for the internalization of the active subunit, CdtB, leading to cell cycle arrest and eventual cell death. Cholesterol also serves as an essential ligand for CdtB, as a CRAC site was identified at 104-VYIYYST-110 (Boesze-Battaglia et al., 2015). Mutations of critical residues in this CRAC site results in decreased cell binding and CdtB internalization in both macrophages and Jurkat cells. Moreover, loss of cholesterol binding capacity led to a concomitant reduction in Cdt induced toxicity; in Jurkats, a loss of cell cycle arrest while in macrophages as decrease in pro-inflammatory responses (Boesze-Battaglia et al., 2015). Collectively, these studies suggest that CdtB and CdtC cholesterol association(s) may also be critical to the mode of action of the toxin, thereby allowing it to hijack lipid raft-associated signaling platform(s) and perhaps provide access to pools of inositol 3,4,5-triphosphate as discussed in detail below.

*Hp*CdtC has an atypical CRAC site where a second valine (V) replaces the central tyrosine (Y) found in the canonical motif. A V77Y mutation in the *Hp*CdtC polypeptide that “restored” the standard CRAC sequence resulted in increased cell toxicity (Zhou et al., 2012). Molecular modeling studies of *Cj*CdtC identified a hydrophobic groove with a CRAC motif consensus sequence and structural properties similar to other Cdt CRAC sites. As with *Aa*CdtC, a mutation of a critical tyrosine residue in the CRAC motif of *Cj*CdtC resulted in decreased *Cj*CDT binding to molecular cholesterol as well as decreased CdtC and CDT holotoxin binding to CHO-K1 cells, decreased nuclear localization and diminished cell cycle arrest (Lai et al., 2013).

## Membrane micro-domains as a signaling platform in Cdt toxicity

The requirement for membrane rafts, sphingomyelin and in some cases direct binding of Cdt to cholesterol suggest that this family of toxins hijacks micro-domain function. In this regard, membrane micro-domain lipids play a major role in cellular signaling. They not only provide the physical constraints that define these membranous regions but often serve as substrate for enzymatic reactions. Generally, signaling lipids have a rapid turnover and are present in minute amounts, although they often transiently appear at high concentrations in subdomains. One of the most well-characterized classes of signaling lipids are the phosphoinositides, these lipids are derived from the phosphorylation of a phosphoinositol head group esterified to two fatty acyl side chains. Phosphatidylinositol can be phosphorylated at its 3, 4, and 5 position in all possible combinations, leading to seven different phosphoinositide species. They serve as a docking site for proteins with domains that recognize specific phosphoinositides (Lemmon, 2008). In addition, the hydrolysis of phosphoinositides yields second messengers that transmit downstream signals. In their docking function, these lipids must be generated at (or targeted to) specific organelles or membrane domains. Therefore, phosphoinositides help define the identity of an organelle or of a domain by recruitment of specific proteins (De Matteis and Godi, 2004). For example, early phagosomes are enriched in PI4,5P2, and PI3,4,5,P3 while secretory vesicles are enriched in PI4P (Billcliff and Lowe, 2014).

The relative level of the individual phosphoinositides is regulated through the coordinated action of kinases and phosphatases. Such spatiotemporal regulation of phosphoinositide production and turnover is critical for proper cell function and relies on the high specificity of these enzymes for particular phosphoinositides. Phosphatidylinositol-3,4,5-triphosphate (PIP3) plays a central role in regulating an array of biological responses; these include cell growth, proliferation and survival, among others (Krauss and Haucke, 2007; Sasaki et al., 2007; Buckler et al., 2008; Huang and Sauer, 2010). PIP3 is normally maintained at low intracellular levels and increases rapidly in response to a variety of signals that involve plasma membrane recruitment. Normal cell function requires that PIP3 levels be tightly regulated; three degradative enzymes, ptase, and tensin homolog deleted on chromosome 10 (PTEN), src homology 2-containing inositol phosphatase 1 and 2 (SHIP1 and SHIP2), have been shown to play critical roles in this capacity (Krystal, 2000; March and Ravichandran, 2002; Seminario et al., 2003). The tumor suppressor phosphatase, PTEN hydrolyses PIP3 to phosphatidylinositol-4,5-biphosphate (PI-4,5-P2). SHIP1 and SHIP2 are inositol 5-phosphatases (IP5P); whereas SHIP2 is ubiquitously expressed, SHIP1 appears to be found in a limited subset of cells including most immune cells. Both SHIP enzymes hydrolyze PIP3 to phosphatidylinositol-3,4-biphosphate (PI-3,4P2) and inositol 1,3,4,5-tetrakisphosphate to inositol 1,3,4-triphosphate.

Sequence and structural homology of CdtBs with members of a metalloenzyme superfamily led to the suggestion that CdtB functions as a phosphoesterase (Dlakić, 2000). Initially these comparisons led investigators to propose that CdtB functions as a DNase, thereby leading to double stranded DNA breaks, activation of DNA repair mechanisms and cell cycle arrest (Cortes-Bratti et al., 2001a,b; Li et al., 2002). More recent analyses suggested that *Aa*CdtB could act as a lipid phosphatase rather than a DNase, as members of this superfamily share the same fold and operate using a select group of strictly conserved catalytic residues (Dlakic, 2001). Indeed, the *Aa*CdtB subunit exhibits phosphatidylinositol-3,4,5-triphosphate (PIP3) phosphatase activity (Shenker et al., 2007). Breakdown product analysis indicates that CdtB hydrolyzes PIP3 to PI-3,4-P2 and therefore functions in a manner similar to phosphatidylinositol 5-phosphatases, with Cdt inducing a time-dependent reduction of PI-3,4,5-P3 in lymphocytes, mast cells and macrophages (Shenker et al., 2014, 2016). When conserved amino acids critical to catalysis were mutated in the *cdtB* gene, the mutant proteins exhibit reduced phosphatase activity along with decreased ability to induce G2 arrest. Lymphoid cells with defects in SHIP1 and/or PTEN (such as Jurkat, CEM, Molt) and, concomitantly, elevated baseline levels of PIP3, were more sensitive to the toxin than HUT78 cells which contain functional levels of both enzymes and low levels of PI-3,4,5-P3. Finally, reduction of Jurkat cell PIP3 synthesis using the PI3K inhibitors, wortmannin and LY290004, protects cells from toxin-induced cell cycle arrest (Shenker et al., 2007). Shenker et al. proposed that *Aa*Cdt toxicity is the result of PIP3 depletion and perturbation of the PI-3K/PIP3/Akt signaling pathway (Shenker et al., 2016). To further delineate the contribution of *Aa*CdtB DNAse activity and phosphatase activity to toxin-mediated toxicity, a series of *Aa*CdtB mutants in which CdtB phosphatase activity was retained but DNAase activity was abolished was analyzed. In both lymphocytes and HeLa cells the ability of toxin to induce cell cycle arrest correlates with retention of phosphatase activity, these cells undergo G2 arrest in the absence of H2AX phosphorylation.

The identification of *Aa*CdtB as a phosphatidylinositol 5-phosphatase coupled with the observation that the holotoxin requires membrane raft integrity to induce toxicity suggest that the lipid associated toxin activities act synergistically to induce maximal toxicity (Calay et al., 2010; Gao et al., 2011). In host cells, the PI3K/Akt signaling pathway is initiated at the plasma membrane where pools of PIP3 are generated by PI3K. A critical consequence of this signaling cascade is recruitment of Akt to the membrane and its phosphorylation through the action of two sequential kinase reactions, one of which is phosphoinositol dependent kinase 1 (PDK1) in a membrane raft requiring process. Fluorescence correlation spectroscopy studies demonstrate that membrane rafts play a critical role in recruiting Akt to the membrane after PIP3 production, with disruption of rafts inhibiting the recruitment process (Calay et al., 2010). Akt activation requires activated PI3 kinase, with the counter-regulatory process utilizing enzyme phosphatases. In this regard, the PTEN-phosphatase activity is also compartmentalized as it localizes outside of membrane rafts. The transient coalescence of membrane domains leads to PTEN's relocation to rafts where phosphatase activity abolishes Akt signaling (Gao et al., 2011). In addition, ceramide induces mislocalization of PTEN to membrane rafts resulting in the inhibition of Akt phosphorylation (Gao et al., 2011). *Aa*CdtB action likely affects multiple effectors, with the cholesterol binding domain targeting the toxin to membrane rafts where the phosphatase activity decreases PIP3 production resulting in inhibition of PDK1 and abolishes pAkt signaling.

In addition to playing a critical role in cell signaling phosphoinositide levels regulate the formation and trafficking of phagocytosed particles (Gillooly et al., 2001; Krauss and Haucke, 2007). For example, PIP3 is formed at the phagosomal cup, and it rapidly disappears after the phagosome has been sealed off from the plasma membrane (Marshall et al., 2001). The disappearance of PIP3 is most likely mediated by lipid phosphatases that are recruited to the newly formed phagosome (Ellson et al., 2001). Identification of the *Aa*CdtB as a lipid phosphatase is consistent with studies which demonstrated that *Aa*Cdt inhibits macrophage phagocytosis (Ando-Suguimoto et al., 2014). We further propose that this inhibition is due to the disruption of the PIP3 rich surface of the phagocytic cup or early phagosome. In addition to a decrease in PIP3 levels, AaCdtB activity increases PI,3,4P2 levels a molecule with known signaling properties. PI,3,4P2 plays a role in the scission of endocytic vessels from the plasma membrane and promoting the recruitment and activation of dynamin and clathrin mediated endocytosis. Thus we can hypothesize that CdtB plays a role in endosome internalization and likely defining the role of early endosomes as sorting (involved in receptor recycling), maturing (fusion with lysosomes), or signaling (Hawkins and Stephens, 2016).

## Toxin internalization and trafficking

**Figure 1:**
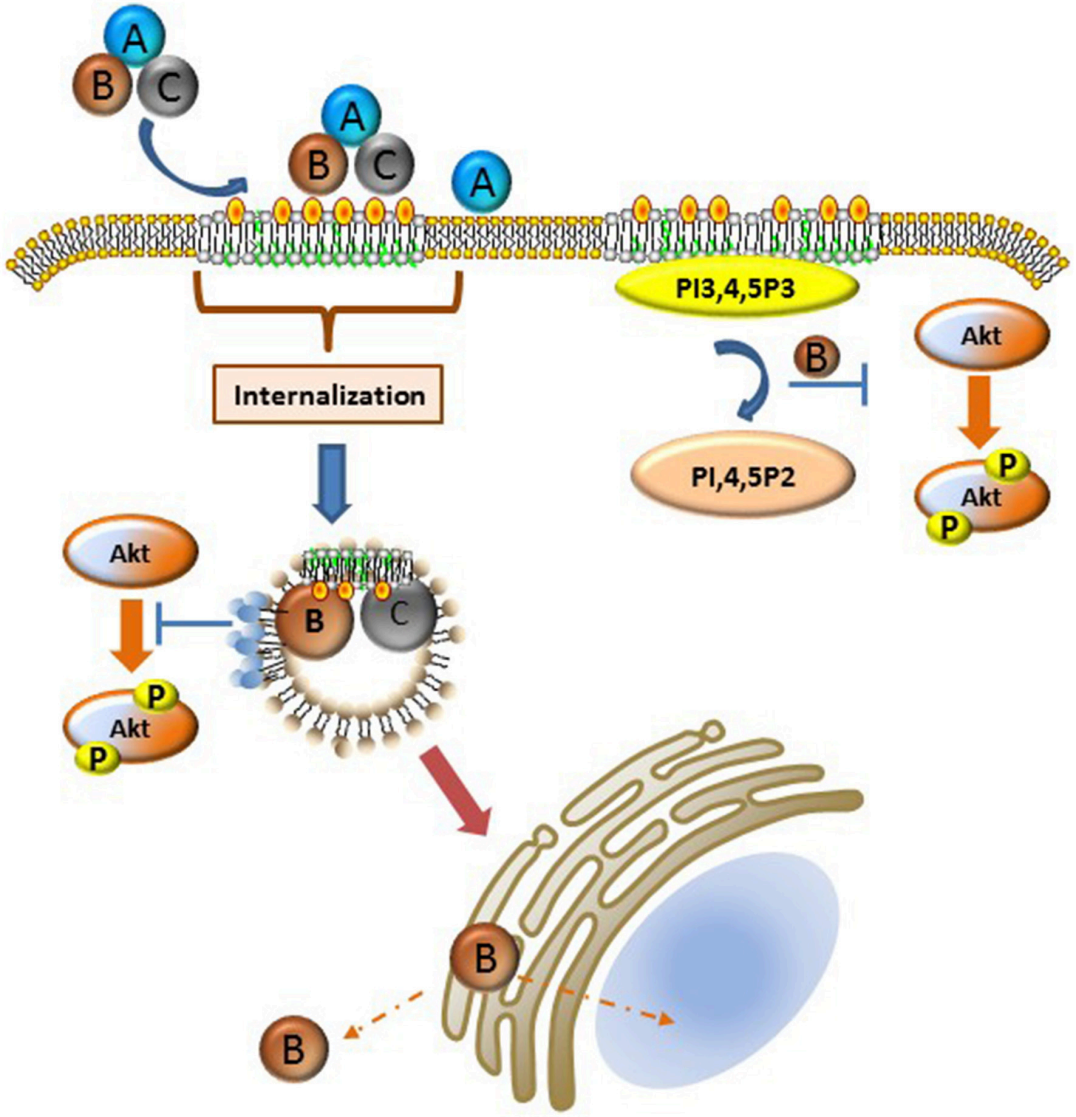
**Schematic representation of Cdt-cell surface association, internalization, and intracellular activity**. Details of these processes are described in the text. Briefly, Cdt holotoxin binds cholesterol rich membrane rafts. Several different pathways have been implicated for the internalization of the CdtB and CdtC subunits; it is possible that the operative pathway may be Cdt sub type specific or host cell specific. Once internalized CdtB traffics to the Golgi, ER, and ultimately to the nucleus and/or cytoplasm. Internalized CdtB exerts toxicity either through its ability to act as a DNase and/or lipid phosphatase converting PI3,4,5,P3 to PI, 4,5P2 leading to PI-3K signaling blockade. Phosphoinositide pools, particularly PIP3, are likely plasma membrane associated, perhaps in the context of membrane rafts or a component of the endosomal substrate pool as indicated by the light blue circles on endosomes which represent PI3,4,5P3. Orange-yellow circles represent cholesterol.

A large number of bacterial toxins affect cytosolic targets in mammalian cells and as such must develop the requisite mechanisms to hijack host cell endocytic and trafficking pathways (for review see Spooner et al., 2006; Watson and Spooner, 2006). In the case of the Cdts, the internalization and trafficking profile is likely the least well-understood aspect of toxin action. These processes appear to share the least homology, suggesting that these pathways are both bacterium species-specific as well as cell type specific. Several fundamental observations provide some unifying themes: 1. Cdts generally bind to cell surface molecules (receptors) likely directly to cholesterol or to component(s) of membrane rafts as discussed above, 2. binding is followed by internalization of the CdtC and CdtB subunits, and 3. one mechanism of Cdt action requires delivery of the CdtB subunit to intracellular components including subcellular compartments such as the nucleus. We will discuss endocytosis and trafficking in the context of the available literature as it relates to membrane bilayer organization at the plasma membrane and within intracellular domains. Our current understanding of these processes is summarized in Figure 1.

### Endocytosis

Several early studies established that Cdt intoxication requires internalization, with CdtC and CdtB localized inside the cell (Akifusa et al., 2005; Shenker et al., 2005; Damek-Poprawa et al., 2012) thus *Aa*CdtC might have a dual role in holotoxin binding and *Aa*CdtB internalization (Mao and DiRienzo, 2002; Akifusa et al., 2005). The X-ray structure of *EcII*CdtB in which its structure alone was compared to its structure as part of the holotoxin complex, suggests that Cdt intoxication requires holotoxin disassembly (Hontz et al., 2006). In a theoretical model, (Guerra et al., 2011) suggest that all three Cdt subunits are internalized by dynamin dependent endocytosis, although they provide no experimental evidence in support of endocytosis of the heterotrimeric complex. Using live cell confocal imaging of FlAsH tagged Cdt subunits in CHO-K1 cells, the Di Rienzo lab showed that *Aa*CdtA localizes exclusively to the cell surface while *Aa*CdtC and *Aa*CdtB are cytosolic; *Aa*CdtB was eventually detected in nuclear fractions by immunoblotting techniques (Damek-Poprawa et al., 2012).

Evidence for an endocytic event comes from studies of *Hd*Cdt and *Aa*Cdt; HEP-2 cells were protected from toxicity when depleted of clathrin or clathrin-coated pit formation was pharmacologically inhibited (Cortes-Bratti et al., 2000). Further evidence supporting Cdts as utilizing the host cell endolysosomal pathways was provided when cells treated with several inhibitors of endosome-lysosome fusion or endosome trafficking were found to be refractory to *Hd*Cdt, *EcII*Cdt, and *Aa*Cdt intoxication (Cortes-Bratti et al., 2000; Dixon et al., 2015). The organization of components into membrane raft domains combined with *Aa*CdtB's action as a lipid phosphatase likely aids in the endocytic process through spatio-temporal localization of toxin to distinct regions on the cells surface and the generation of PI3,4P2 known to be critical in the formation of early endosomes (Mayinger, 2012; Hawkins and Stephens, 2016).

### Trafficking-Cdts utilize aspects of retrograde transport

CdtB internalization was also shown to be dynamin dependent in HeLa cells. Dynamin, a GTPase, polymerizes, aiding in the formation of an endosomal vesicle (Guerra et al., 2011; DiRienzo, 2014). Disruption of golgi integrity by bafilomycin A, an inhibitor of the H(+)-ATPase prevented *Hd*Cdt intoxication of HeLa cells, prompting several investigators to suggest that Cdts hijack host retrograde transport pathways. Experimental evidence in support of this hypothesis was provided using *Hd*CdtB mutants that were engineered to contain a Golgi mediated sulfation site as well as an ER dependent glycosylation site. In these studies, the *Hd*CdtB subunit was observed to be both sulfated and glycosylated (Guerra et al., 2005) suggesting retrograde transport of CdtB from Golgi to the endoplasmic reticulum (ER). Similar studies have not been undertaken for the other Cdts. Intracellular trafficking may also be regulated at the level of membrane raft association as the *Aa*CdtB cholesterol binding domain may contribute to its trafficking. Intracellular membrane raft-like complexes are becoming increasingly well-characterized; for example, the trans-Golgi is enriched in GM1, cholesterol, PI, and Rab proteins. *Hd*Cdt and *Ec*Cdt localization was found to depend on Rab 7 with Rab9 colocalization, (Gargi et al., 2012; Dixon et al., 2015) both of which are regulated by cholesterol thereby contributing *in vivo* to endosome motility (Chen et al., 2008). Lastly, as illustrated in Figure 1, the endosomal pool of PI,3,4,5P3 may serve as substrate for *Aa*CdtB thereby inhibiting the localized activation of Akt (Jethwa et al., 2015).

Currently, there is little agreement on the route by which CdtB leaves the ER; some studies suggest that CdtB translocates out of the ER using an endoplasmic reticulum degradation pathway (ERAD) dependent pathway. Eshraghi et al. (2014) showed that deletion of critical components of the ERAD degradation pathway, derlin (derl2), Hrd1, and p97 rendered CHO cells resistant to *Aa*Cdt and *Hd*Cdt and lead to the retention of *Hd*CdtB in the ER. Previously identified domains within DerL2 required for ERAD of misfolded proteins are not utilized by the Cdts. However, two previously uncharacterized domains within Derl2 are required for intoxication by *Aa*Cdt and *Hd*Cdt as well as by ricin.

Based on the generality that most proteins require at least partial unfolding to move from the ER to the cytosol, (Guerra et al., 2009), found that of the three *Hd*Cdt subunits, *Hd*CdtB was found to be the most thermally stable. In addition, when HeLa cells were pretreated with the chemical chaperone, glycerol, known to inhibit protein unfolding there was no change in Cdt mediated H2AX phosphorylation. These authors predicted that *Hd*CdtB likely uses an ERAD independent mechanism to translocate to the nucleus. They however did not address if CdtB mediated toxicity could be due to the phosphatase activity previously observed with *Aa*CdtB, a function that does not require nuclear translocation.

How and whether nuclear translocation of CdtB takes place is still an open question. If CdtB's main physiological mode of action is as a DNAase, it would have to find its way to the nucleus in order to exert its function. In this regard, both *EcII*CdtB and *Aa*CdtB contain nuclear localization signals (NLSs). *Aa*CdtB contains an atypical NLS which appears to be essential for nuclear localization and cell intoxication (Nishikubo et al., 2003). *EcII*CdtB contains two NLS, deletion of which prevents *EcII*CdtB nuclear localization and cell cycle arrest but has not effect on DNAse activity (Mcsweeney and Dreyfus, 2004).

## Summary

A cumulative assessment of the data regarding Cdts suggests that they are capable of utilizing different aspects of the lipidome at various stages of host–pathogen interactions. Several members of this toxin family hijack host cell pathways via membrane rafts by exploiting the properties of these micro-domains; they are highly dynamic entities that fuse, stir, and continuously modify their shape. These plasma and intracellular membrane domains have often been compared to a myriad of mercury sheets perpetually moving (Taieb et al., 2004). Viruses, bacteria, and toxins bind various constituents of these domains thereby curtailing the dynamic nature of these structures. We propose that *Aa*Cdt association with cells results in a redistribution of cell surface micro-domains with the direct binding of *Aa*CdtC and *Aa*CdtB likely clustering the toxin on the cell surface and inhibiting transient rearrangement of the rafts. This reorganization favors endocytosis resulting in the internalization of the CdtB/CdtC toxin subunits, as illustrated schematically in Figure 1. The lipid phosphatase activity of the CdtB enzymatically modifies lipid components such that signaling platforms are altered with a shift in the phosphoinositide pool. A consequence of this alteration in membrane bilayer organization is loss of Akt signaling due to the depletion of the PI3K pool and the inability of raft domains to coalesce to a form an active signaling platform. Thus the two independent lipid binding/activity regions in CdtB, a cholesterol binding domain and a lipid phosphatase active site function cooperatively to induce toxicity and alter host regulatory responses. Investigations focusing on the species specificity of this activity and its relationship to intracellular trafficking and cell cycle arrest are necessary to unveil CDT-host interactions at the molecular level.

## Author contributions

Conception or design of work: KB, BS, DA, and MD. Draft and revisions of this article critically evaluated for important intellectual content: KB, DA, BS, and MD. Final approval: KB, DA, BS, and MD.

### Conflict of interest statement

The authors declare that the research was conducted in the absence of any commercial or financial relationships that could be construed as a potential conflict of interest.
